# Exploring the impact of the COVID-19 pandemic on healthcare and substance use service access among women who inject drugs: a qualitative study

**DOI:** 10.1186/s12954-023-00793-y

**Published:** 2023-06-09

**Authors:** Lyra Cooper, Joseph G. Rosen, Leanne Zhang, Danielle Pelaez, Praise F. Olatunde, Jill Owczarzak, Ju Nyeong Park, Jennifer L. Glick

**Affiliations:** 1 Department of Health, Behavior, and Society, Bloomberg School of Public Health, Johns Hopkins University, Baltimore, MD USA; 2 Department of International Health, Bloomberg School of Public Health, Johns Hopkins University, Baltimore, MD USA; 3 Division of General Internal Medicine, Warren Alpert Medical School, Brown University, Providence, RI USA; 4 Center for Biomedical Research Excellence on Opioids and Overdose, Rhode Island Hospital, Providence, RI USA; 5 Oakland, USA

**Keywords:** COVID-19, Substance use, Women who inject drugs, Healthcare access, United States

## Abstract

**Background:**

The COVID-19 pandemic disrupted healthcare and substance use services engagement, including primary and mental health services as well as residential and outpatient drug treatment. Women who inject drugs (WWID) face known barriers to healthcare and substance use service engagement, which pre-date the COVID-19 pandemic. The impact of COVID-19 on WWID’s engagement with healthcare and substance use services, however, remains understudied.

**Methods:**

To explore the impact of the COVID-19 pandemic on service-seeking and utilization, we conducted in-depth interviews with 27 cisgender WWID in Baltimore, Maryland, in April–September 2021. Iterative, team-based thematic analysis of interview transcripts identified disruptions and adaptations to healthcare and substance use services during the COVID-19 pandemic.

**Results:**

The COVID-19 pandemic disrupted service engagement for WWID through service closures, pandemic safety measures restricting in-person service provision, and concerns related to contracting COVID-19 at service sites. However, participants also described various service adaptations, including telehealth, multi-month prescriptions, and expanded service delivery modalities (e.g., mobile and home delivery of harm reduction services), which overwhelmingly increased service engagement.

**Conclusion:**

To build upon service adaptations occurring during the pandemic and maximize expanded access for WWID, it is vital for healthcare and substance use service providers to continue prioritizing expansion of service delivery modality options, like telehealth and the provision of existing harm reduction services through alternative platforms (e.g., mobile services), that facilitate care continuity and increase coverage.

## Background

Women who inject drugs (WWID) face unique health challenges compared to their male counterparts. Studies have shown that WWID experience elevated risk of sexually transmitted infections, violence, and trauma relative to men who inject drugs and are disproportionately impacted by gender-based violence and trauma [1, 2]. Gendered power dynamics may also amplify substance-related harms (if women inject after their partners or require injection assistance) and HIV/STI infection rates [3–5].

WWID encounter overlapping barriers to accessing healthcare and substance use services, including residential and outpatient drug treatment services, counseling and mental health services, prescription refills, and syringe exchange [6, 7]. Women are more likely than their male counterparts to experience economic and temporal barriers to accessing and engaging with treatment (e.g., lack of time to attend treatment services due to familial responsibilities, transportation challenges), and to experience severe anxiety, depression, mood disorders, and substance use stigma, which subsequently decrease their likelihood of service-seeking and care continuity [6, 8–10]. These data demonstrate the gendered differences in healthcare-seeking for substance use services among WWID [6, 10, 11].

The COVID-19 pandemic has not only increased rates of substance use, but it has exacerbated the pre-existing access and engagement barriers and health challenges faced by WWID [12]. For instance, syringe service programs (SSPs) in some U.S. jurisdictions suspended their operations, preventing women from utilizing crucial healthcare and harm reduction services [13, 14]. While some healthcare and substance use service programs remained open, they were required to adapt their existing service delivery infrastructure to minimize person-to-person contact (e.g., take-home methadone, telehealth behavioral health services), disrupting client daily routines, increasing social isolation, and exacerbating access inequities for healthcare and ancillary services [15, 16].

Despite obvious negative implications of service disruptions observed at the start of the COVID-19 pandemic, these very disruptions also catalyzed innovation of healthcare and substance use service delivery that facilitated continuity of care and, in some cases, increased equity in service provision [14, 17, 18]. One significant innovation was telehealth, which has allowed providers to care for patients over the phone, through online secure video chats or messaging, and via remote monitoring [19]. During the pandemic, telehealth allowed U.S. substance use service providers to engage and retain patients receiving medication for opioid use disorder (MOUD) due to temporary changes in federal regulations (i.e., Drug Enforcement Administration registered practitioners were able to prescribe controlled substances without an in-person visit) [16, 20–22]. Moreover, some SSPs were able to limit their pandemic closure time by designating themselves as a rapid essential service, adapting by providing delivery and mail-based supply distribution and telehealth services [14, 17, 18]. Through these adaptations and innovations, some healthcare and harm reduction service providers were able to maintain and even increase access to previously unreached populations.

Given the known disruptive impacts of the COVID-19 pandemic on healthcare and substance use service provision broadly, coupled with pre-existing service access barriers faced by WWID, it is likely that the pandemic exacerbated access and engagement barriers for this population. Nevertheless, studies have yet to investigate how WWID navigated pandemic-related disruptions to healthcare and substance use services, as well as their experiences with service delivery modifications at the start of the COVID-19 pandemic [11, 23].

This study aims to better understand the impact of the COVID-19 pandemic on healthcare and substance use service provision, access, and engagement among cisgender WWID in Baltimore, Maryland, as well as WWID’s perspectives on seeking and utilizing healthcare and substance use services modified at the start of the pandemic.

## Methods

Data for this study were collected as part of Optimizing HIV Pre-Exposure Prophylaxis (PrEP) among Women Living in Baltimore City (OPAL), a multi-phase formative research study aiming to develop and pilot a strategy for enhancing PrEP engagement among WWID in Baltimore, Maryland [24–26].

We purposefully recruited WWID for telephone interviews through flier dissemination at local harm reduction organizations and for in-person interviews outside of these organizations via street intercept. Between April and September 2021, we interviewed 27 participants. Our inclusion criteria consisted of: (1) cisgender women (people assigned female at birth who identify as women), (2) aged 18 years or older, (3) self-reported injection of any drugs not prescribed by a provider in the past 6 months, and (4) English-speaking. Women who self-reported living with HIV were excluded given their ineligibility for PrEP; transgender women were ineligible, as the focus of the parent study was to identify constraints and opportunities for PrEP engagement among cisgender women [24–26]. Moreover, transgender and cisgender women have different needs and barriers requiring separate studies or a study that is resourced to investigate both [27]. After providing verbal informed consent, participants completed a brief interviewer-administered demographic questionnaire and an in-depth, semi-structured interview in-person (*n* = 19) or via telephone (*n* = 8). The interviews covered a range of topics including health service engagement, daily needs, PrEP awareness/perspectives, and the impact of the COVID-19 pandemic on healthcare and substance use service engagement. Interviews lasted between 30 and 60 min and were audio-recorded. Participants received $50 prepaid Visa cards for completing study activities.

Interview recordings were professionally transcribed and quality checked by interviewers for readability, comprehension, accuracy, and redaction of personally-identifying information. We developed a codebook through an iterative, team-based thematic analysis of salient concepts emerging from the transcripts. First, we identified a list of topics from the interview guide to act as preliminary organizing themes. Then, we implemented open coding, reading interview transcripts line-by-line to discern new themes and codes to include within the burgeoning codebook. We collectively condensed open codes into focused codes, and then grouped these focused codes within overarching thematic categories [28]. Two study team members (LC, PFO) piloted the initial codebook, independently applying codes to a subgroup of transcripts, and then a third member reviewed the code applications and identified discrepancies (JGR). After finalizing the codebook, we uploaded transcripts into Dedoose 9.0 (SocioCultural Research Consultants, Manhattan Beach, CA). Each transcript was coded independently by one study team member (LC, LZ, PFO), and all coded transcripts were reviewed by a study manager (JGR) for fidelity to the codebook.

After coding all transcripts, we generated code reports (i.e., text segments tagged to specific codes) focusing on the COVID-19 pandemic’s impact on healthcare and substance use services. After reviewing code reports, we re-grouped text segments into themes and sub-themes, reviewed analytic memos, and ultimately organized the data under two overarching domains: (1) pandemic impact on provision of, access to, and engagement with healthcare and substance use services, and (2) service delivery innovations to address disruptions to healthcare and substance use services. We present study findings in narrative form under these two overarching concepts below, with illustrative quotes.

## Results

### Participant characteristics

Table 1 summarizes demographic and substance use characteristics of interviewed WWID (*N* = 27). The average age of participants was 39 years (range: 21–60 years); a majority of participants identified as White (59%) or Black (26%), with some identifying as American Indian or Alaska Native (7%) or multiracial (7%). Polysubstance use was high among women in our study (96%), with 96% using opioids, 93% stimulants, 74% non-prescribed painkillers, 74% club drugs (MDMA, Rohypnol, GHB), 52% alcohol, and 33% marijuana. Overall, 78% injected drugs in the past month and 71% injected daily.

**Table 1:** Demographics and characteristics of cisgender women who inject drugs in Baltimore City, Maryland participating in semi-structured, in-depth interviews (*N* = 27)

Characteristics	*n*	%
Race		
White	16	59.3
Black/African American	7	25.9
American Indian or Alaska Native	2	7.4
Multiracial	2	7.4
Substance use (past 30 days)		
Opioids	26	96.3
Stimulants	25	92.6
Non-prescribed painkillers	20	74.1
Club drugs	20	74.1
Alcohol	14	51.9
Marijuana	9	33.3
Polysubstance use	26	96.3
IDU in the past month	21	77.8
Daily IDU frequency	15	71.4

Club drugs = MDMA (ecstasy), rohypnol, and GHB); IDU = injection drug use; Daily IDU frequency = percentage of women who inject drugs daily [15] among those who have injected within the past month [21]

### Pandemic-related service delivery disruptions

Due to the pandemic, many women described how healthcare and substance use services were forced to suspend their operations, reduce the availability of in-person care, and implement pandemic safety measures. For instance, due to pandemic-related service closures, some women reported being unable to access peer support groups, which are crucial for some WWID with substance use disorders.

Places are closed, and you can't get people on the phone, and it's been very aggravating…I know that during the pandemic and still, there are no AA [alcoholics anonymous] or NA [narcotics anonymous] meetings.

Participants also described decreased availability of in-person healthcare services, including primary care.

My primary care provider actually wasn’t seeing people in the office. They wanted to do a virtual appointment. It seems like it’s a longer wait time, and you can’t have people with you, or you can’t go with people, so it seems like people are less likely to go.

Even when services continued being available in-person, women cited the declining staff capacity as another consequence of the COVID-19 pandemic.

There’s only two doctors on the van [mobile health clinic], and now, there’s only the one that I see. She’s the only one doing it because the other one went to go work with COVID patients at the hospitals...​​She [provider] has a whole caseload to herself, so she can’t do everything that she needs to be doing.

This diminishing capacity also included reduced operational hours. Participants reported difficulty scheduling appointments with healthcare providers, given some providers were unable to accept new patients. One woman reflected on her experience engaging with services from urgent care, which was her primary source of healthcare.

A lot of times, the hours are shorter. There’s been a lot of places that are closed.

In addition to reduced operational capacity and service closures, participants highlighted how providers instituted a myriad of pandemic safety measures to protect staff and patients from contracting COVID-19. Consequently, women described how these measures discouraged them from seeking out and obtaining needed substance use services. One participant highlighted COVID-19 symptom screening as a barrier to utilizing available in-person care.

Just to be able to get in the building…here, they want you to fill out this whole 10-page thing about how you’ve been feeling in the past 3 days. It’s overwhelming.

Another participant explained how healthcare facility efforts to prevent COVID-19 transmission among patients prevented her from accessing detoxification and inpatient drug treatment services during the pandemic.

I wanted to go to rehab out of state. I wanted to really take it seriously and try to change my life, and it has been so hard to get help because…they expect you to go to detox…before you come to them. With COVID, there's so many places that don't want you in a hospital setting because of… the damage that it could do being in there… The pandemic has been awful as far as us trying to get help if you're serious about it.

Lastly, and somewhat in contrast to the above concerns about safety measures impeding engagement, some participants discussed how their concerns about contracting COVID-19 also demotivated care-seeking.

Now that this pandemic come on, nobody really wants to go sit in a hospital because lots of people go there and wind up coming home, and they wind up having it [COVID-19].

### Pandemic service delivery innovations

While many service delivery modifications reduced access and engagement, participants also reported that healthcare and substance use service providers implemented innovations to continue offering low-barrier services to WWID and other marginalized populations. These adaptations included telehealth, multi-month prescriptions, and other novel service delivery modalities.

The transition to telehealth during the COVID-19 pandemic largely expanded care access for the WWID in our study. Participants nearly unanimously identified telehealth as a facilitator of service accessibility and engagement during the pandemic, largely due to the ability to engage in conversations with their providers from convenient locations through phone service or wireless internet. One woman reflects on the convenience of telehealth for psychiatric care.

I can do it anywhere. I don’t have to go specifically to an office. I can be sitting in my car, or I can be absolutely anywhere and do a Zoom [telehealth consultation].

However, for some women, the prospect of telehealth was insufficient to overcome pre-existing barriers to care, which were exacerbated during the pandemic. As a result, timeliness and frequency of interactions with service providers declined for some WWID.

I’ve spoken to [name of nurse redacted] on the phone, but I haven’t actually had a need to see her. But when I did, it was not direct. I couldn’t go to the location, so, less frequent…Definitely a little more difficult having to call or text the phone number that they give you and then waiting to be put in touch with her because she’s not on the phone line. It took maybe a couple days as opposed to you walk in and instantly you can talk.

Alongside telehealth, participants detailed how service providers adopted new prescription practices during the first year of the pandemic, enabling women to obtain larger-quantity MOUD prescriptions (i.e., month-long instead of week-long MOUD prescriptions) with ease over the phone and through video platforms.

When the pandemic first started, they [suboxone program]...told everybody, “You got to call this number.” When we called the next week, they gave us monthly prescriptions. It was just like “bam”, switched over…I prefer a monthly [prescription]…It’s so much easier just getting a month’s worth.

Lastly, WWID shared how harm reduction organizations improved access for them by expanding mobile services (e.g., home delivery). Despite pandemic-related challenges, participants highlighted how these organizations continued to supply harm reduction resources across Baltimore, adapting to pandemic-era service provision restrictions by delivering injection equipment to individuals’ homes and overriding previous one-for-one policies on syringe exchange.

Supplies just get dropped off at the house. It’s a big help. I haven’t been to the needle exchange bus pretty much since the pandemic started because I know at the very beginning, they had shut down. But even since they started back up, I haven’t been. I haven’t had to go because of the deliveries.

They used to give you tool-for-tool, so that if you bring back 10, you get a bag of 10. Now you bring back 10, you get a box of 100.

## Discussion

We sought to better understand the impact of the COVID-19 pandemic on healthcare and substance use service access and engagement among cisgender WWID in Baltimore, Maryland, a marginalized population with well-documented service access constraints predating the COVID-19 pandemic. Participants described multiple service delivery disruptions (closure, decreased in-person service capacity attributed to mitigation measures, concerns regarding COVID-19 transmission), which impeded their care-seeking and utilization of essential health and harm reduction services. Simultaneously, they described various service delivery innovations (telehealth, larger-quantity prescriptions, home delivery of injection equipment), which generally increased accessibility of disrupted services during the COVID-19 pandemic and, in some cases, improved their care experiences. Our findings regarding disruptions to access and engagement align with results from studies of other marginalized populations during the COVID-19 pandemic. For instance, people who use drugs (PWUD) in the Northeastern U.S., Canada, and rural Oregon also experienced diminished access to healthcare and harm reduction services. These studies linked service access barriers to increased risk factors, such as riskier injection practices; increased drug use, supply sharing, and overdose; decreased interest in receiving substance use treatment; and increased housing and food insecurity [23, 29–31]. Our findings, coupled with the extant literature, point to a need for policy and programming that minimize disruptions to healthcare and substance use service provision, especially during future public health crises.

While many WWID experienced disruptions, they also reported service delivery innovations in response to the COVID-19 pandemic, consistent with other study findings. For instance, during the pandemic, Healthcare on the Spot—a Baltimore street-based mobile clinic providing MOUD and infectious disease treatment to impacted communities—adapted to also provide services via telehealth. A larger proportion of the new telehealth clients were female and white as opposed to before the pandemic, highlighting the increased accessibility of MOUD treatment via telehealth modalities for women, as well as existing racial disparities in access and engagement, given most Baltimore residents are Black [16]. However, in our study, telehealth alone insufficiently overcame access barriers for some WWID, and, in some cases, introduced new barriers, like reducing the timely accessibility of services and the frequency of interactions with those services. This is likely due to pre-existing socioeconomic vulnerabilities that were exacerbated by the pandemic, including WWID’s lack of access to telehealth equipment (e.g., phone, computer), reliable internet connection, and digital literacy [32–34]. Nonetheless, telehealth has transformed the healthcare and substance use service landscape, and developing methods to overcome common barriers faced by WWID will be an important area of future implementation research.

WWID in our study also reported expanded harm reduction services during the pandemic, which indicates that Baltimore-based service providers were able to quickly adapt and innovate their programs to meet client needs. For instance, Healthcare on the Spot was able to extend prescription durations by up to 4 weeks, extending visit intervals and, subsequently, improving client retention outcomes [16]. However, this finding diverges from experiences in other settings where PWUD reported increased difficulty accessing injection equipment, appointments with doctors or HIV counselors, lab or blood testing, and sessions with case managers [35–37]. The limited, protracted disruptions to services observed in our study population may be attributable to the constellation of accessible, trusted harm reduction service providers in Baltimore that provide daily support services to PWUD. Women in our study called attention to a need to expand and invest in innovative service delivery models that better meet the needs of marginalized populations like WWID, especially in times of prolonged crisis and uncertainty. Similarly, providers and policymakers have underscored a need to maintain policy changes that occurred during the pandemic and expanded access to services (e.g., telehealth provision of larger-quantity, take-home MOUD prescriptions), even after the peak of the COVID-19 pandemic [38, 39].

Taken together, these study findings have key implications for federal and state policy decisions that sustain innovations to healthcare and harm reduction delivery. These innovations increase accessibility to needed services and allow for additional resources to enhance health system resilience and ensure continuity of healthcare and substance use services. This is especially critical for WWID, who may experience access and engagement barriers more acutely than men due to cost, time limitations, mental health challenges, and heightened stigma associated with substance use and treatment [6, 8, 9, 40]. With these patient-centered policies and innovations in place, harm reduction service and healthcare providers may be better equipped to provide key services in today’s healthcare landscape and pivot to innovative service delivery strategies during times of crisis and beyond, which can facilitate care continuity and, in some instances, increase service delivery equity.

This study has several limitations to consider. First, we pivoted our recruitment and interview format from telephone screenings and interviews to in-person data collection, given outstanding recruitment challenges using telephone-based methods. Data collected from participants in-person (*n* = 19) versus over the phone (*n* = 8) may, therefore, represent different strata of WWID, and we may have not reached saturation on key experiences and themes within each of these groups. Second, our recruitment strategy involved distributing flyers to and intercepting women near harm reduction organizations; therefore, we likely undersampled women not regularly engaging with these services, whose perspectives and experiences may have differed from those of our study population. Third, we relied on interviews with WWID and did not leverage other populations for thematic triangulation and enhancement, including service providers, who may have shared additional perspectives on service adaptations during the COVID-19 pandemic. Additionally, exclusive sampling of cisgender women limited our ability to conduct cross-gender analysis; however, given the dearth of research on WWID, a focus on this population was warranted. Fourth, in-person in-depth interviews were conducted in the summer months of 2021, at which time high heat and humidity rendered data collection physically taxing for study staff and participants alike. These external factors, therefore, may have impacted the potential richness of those interviews. Lastly, we conducted interviews in mid-to-late 2021, nearly 1 year after the onset of the COVID-19 pandemic; participants might have experienced challenges recalling service adaptations and disruptions occurring months prior.

## Conclusion

This study contributes to the rapidly growing literature concerning the impact of the COVID-19 pandemic on the U.S. healthcare system and urban healthcare and substance use service accessibility and engagement among marginalized populations, including WWID. Women in our study faced various healthcare and substance use service access challenges during the pandemic, pointing to the need for increased provider and health system emergency preparedness. Furthermore, participants reported service delivery innovations that facilitated care continuity and, in some cases, expanded the accessibility of healthcare and substance use services. Our study findings reaffirm calls for more innovative service delivery models that facilitate service provision during public health emergencies and preferential expansion of access for the most marginalized populations, like WWID. Our results also reinforce the urgency with which telehealth services and inputs (e.g., broadband WiFi) should be broadly expanded, alongside investments in novel approaches for delivering substance use services (i.e., mobile syringe services, take-home MOUD). This study provides valuable insights regarding the need to enhance and invest in innovative modalities to ensure marginalized populations like WWID can access and utilize healthcare and substance use services while managing other competing survival priorities, during and beyond acute public health crises.

## Data Availability

The datasets generated and/or analyzed during the current study are not publicly available due to the sensitive nature of the data but are available from the corresponding author on reasonable request.

## Abbreviations

MOUD: Medication for opioid use disorder
OPAL: Optimizing HIV pre-exposure prophylaxis among women living in Baltimore City study
PrEP: Pre-exposure prophylaxis
PWUD: People who use drugs
SSPs: Syringe service programs
WWID: Women who inject drugs
